# Prediction in Autism by Deep Learning Short-Time Spontaneous Hemodynamic Fluctuations

**DOI:** 10.3389/fnins.2019.01120

**Published:** 2019-11-08

**Authors:** Lingyu Xu, Xiulin Geng, Xiaoyu He, Jun Li, Jie Yu

**Affiliations:** ^1^Shanghai Institute for Advanced Communication and Data Science, Shanghai University, Shanghai, China; ^2^School of Computer Engineering and Science, Shanghai University, Shanghai, China; ^3^Guangdong Provincial Key Laboratory of Optical Information Materials and Technology, South China Academy of Advanced Optoelectronics, South China Normal University, Guangzhou, China; ^4^Key Lab for Behavioral Economic Science & Technology, South China Normal University, Guangzhou, China

**Keywords:** ASD, fNIRS, neural network, time series, CGRNN model

## Abstract

This study aims to explore the possibility of using a multilayer artificial neural network for the classification between children with autism spectrum disorder (ASD) and typically developing (TD) children based on short-time spontaneous hemodynamic fluctuations. Spontaneous hemodynamic fluctuations were collected by a functional near-infrared spectroscopy setup from bilateral inferior frontal gyrus and temporal cortex in 25 children with ASD and 22 TD children. To perform feature extraction and classification, a multilayer neural network called CGRNN was used which combined a convolution neural network (CNN) and a gate recurrent unit (GRU), since CGRNN has a strong ability in finding characteristic features and acquiring intrinsic relationship in time series. For the training and predicting, short-time (7 s) time-series raw functional near-infrared spectroscopy (fNIRS) signals were used as the input of the network. To avoid the over-fitting problem and effectively extract useful differentiation features from a sample with a very limited size (e.g., 25 ASDs and 22 TDs), a sliding window approach was utilized in which the initially recorded long-time (e.g., 480 s) time-series was divided into many partially overlapped short-time (7 s) sequences. By using this combined deep-learning network, a high accurate classification between ASD and TD could be achieved even with a single optical channel, e.g., 92.2% accuracy, 85.0% sensitivity, and 99.4% specificity. This result implies that the multilayer neural network CGRNN can identify characteristic features associated with ASD even in a short-time spontaneous hemodynamic fluctuation from a single optical channel, and second, the CGRNN can provide highly accurate prediction in ASD.

## Introduction

Autism spectrum disorder (ASD) refers to a group of neurodevelopmental disorders, including autism and Asperger’s syndrome (AS). The current diagnostic criteria for ASD focus on two core symptoms: social communication impairment, restricted interests, and repetitive behaviors (Sharma et al., 2018). Due to the complexity and diversity of ASD, it often takes a long time from detection of the behavioral signs to the definitive diagnosis, which inevitably leads to the lagging of necessary treatment or intervention. In recent years, the ASD prevalence is increasing rapidly (e.g., 1 in 59, with the prevalence of 4:1 male to females), therefore the study in ASD has drawn significant attention to the public (Christensen et al., 2016). To overcome the drawback that the diagnosis of ASD relies on behavioral observation solely, a variety of studies have been performed, including those brain imaging studies to find characteristics associated with this disorder. On the other hand, with the advance of machine learning, in particular, deep-learning artificial neural network, it may become possible for neurologists to use these machine-learning algorithms to analyze the brain image data collected from ASD and perform image-based early diagnosis of ASD. In addition to this, machine-learning may also play a promising role in ASD intervention, for instance, using personalized intelligent robots to interact with ASD individuals to improve their behaviors (Amaral et al., 2017; Rudovic et al., 2018).

A large variety of brain image studies have demonstrated functional and structural abnormalities in brains of ASD. For example, magnetic resonance imaging (MRI) studies have uncovered that individuals with ASD present an aberrant age-related brain growth trajectory in the frontal area (Elizabeth and Eric, 2005; Lainhart, 2010; Courchesne et al., 2011), which strongly suggests that functional brain measurement at young ages is crucial for revealing ongoing abnormalities in ASD. Libero et al. (2015) utilized multimodal brain imaging modalities [structural MRI, diffusion tensor imaging (DTI), and hydrogen proton magnetic resonance spectrum (1H-MRS)] to investigate neural structure in the same group of individuals (19 adults with ASD and 18 adults with TD) and used the decision tree with fractional anisotropy (FA), radial diffusivity (RD), and cortical thickness as features to perform classification between ASD and TD. This combination method overcomes the discrepancy problem arising from using each imaging method separately (Libero et al., 2015). Some functional brain studies have shown atypical brain activation in response to various cognitive tasks or decreased resting-state functional connectivity (RSFC). These characteristics could also be used for differentiating between individuals with ASD and TD individuals (Kaiser and Pelphrey, 2012; Murdaugh et al., 2012; Deshpande et al., 2013). For example, Iidaka calculated the correlation matrix of resting-state functional magnetic resonance imaging (RS-fMRI) time series and then sent the matrix as input to a probabilistic neural network (PNN) for the classification, which demonstrated that the inherent connection matrix generated by RS-fMRI data might serve as biomarkers for predicting ASD (Iidaka, 2015).

Functional near-infrared spectroscopy (fNIRS) as an optical brain imaging modality utilizing near-infrared light to probe human brain functional activity, is advancing rapidly in techniques and applications. Hong et al. (2018) investigated a brain-computer interface framework for hybrid fNIRS and electroencephalography (EEG) for locked-in syndrome (LIS) patients, and found that the prefrontal cortex is identified as a suitable brain region for imaging. They also studied hybrid fNIRS and EEG for early detection of hemodynamic responses (Hong and Khan, 2017; Khan et al., 2018). Furthermore, they developed a new vector phase diagram to differentiate the initial dip phase and the delayed hemodynamic response (HR) phase of oxy-hemoglobin changes (ΔHbO) (Zafar and Hong, 2018). Very recently fNIRS was also adopted in the investigation of atypical brain activity associated with ASD (Adelina and Bravo, 2011; Jung et al., 2016; Li and Yu, 2018). For instance, Mitsuru et al. measured brain hemodynamic fluctuations of bilateral Brodmann area 10 (BA10) in 3- to 7-year-old ASD and TD children under conscious conditions. They found that slow hemodynamic fluctuations showed abnormal functional connections in ASD (Mitsuru et al., 2013).

Thus far, most of the classifications between ASD patients and normal controls depend on prior characteristic features extracted empirically from brain images. However, due to the complexity and limited knowledge about the pathogenic mechanism of ASD, the hidden factors associated with ASD, which can be used for accurate differentiation between ASD and normal controls, are not easy to be observed and identified merely through reading the brain images. Since the deep-learning artificial neural network is a data-driven method, has the ability to find characteristics hidden in the complete data set. We hypothesize that deep-leaning model might be used for the prediction of ASD through brain images, in particular, our fNIRS data collected from children with ASD, though deep learning based approaches have not been well studied (Ilias et al., 2016; Dvornek et al., 2017; Chiarelli et al., 2018).

On the other hand, a critical challenge for acquiring brain images of most of brain imaging modalities such as MRI/functional magnetic resonance imaging (fMRI), magnetoencephalography (MEG), single photon emission computed tomography (SPECT) and positron emission tomography (PET), et al. is that the subject has to be strictly still during image acquisition that could last 5–10 min or longer. It is not an easy task for conscious (not sedated) children, in particular children with ASD. Therefore if the characteristics of ASD can extract from brain images collected in a short time, it is of great practical significance for brain imaging study in ASD. Even though EEG and fNIRS are not as sensitive to motion as those imaging techniques mentioned above, the artifact caused by head movement still can deteriorate the time-series signals, resulting in an inaccurate result. Thus we aim at two goals in this study: (1) exploring the possibility of using a deep-learning neural network to extract features associated with ASD from fNIRS signals; and (2) using a short-time (e.g., 7 s) fNIRS time series to perform accurate classification between ASD and TD children.

To test our hypothesis and realize the goals, we collected approximate 8-minute spontaneous hemodynamic fluctuations from the bilateral inferior frontal gyrus and temporal lobe by an fNIRS setup in 25 children with ASD and 22 TD children. To analyze the fNIRS data [i.e., time-series of oxygenated hemoglobin (HbO_2_), deoxygenated hemoglobin (Hb), and total hemoglobin (HbT = HbO_2_ + Hb)], we designed a multilayer neural network consisting of CNN and GRU as a combined unit (called CGRNN) for learning and predicting ASD. The CGRNN is powerful in recognizing characteristic features, identifying the relationship among data in the sequence, and has low computation cost. To test the possibility of using short-time spontaneous hemodynamic fluctuations for the differentiation between ASD and TD, we segregated the long-time (i.e., 8 min) data sequence into many overlapped short-time (i.e., 7 s) sub-sequences, and then sent them as the input to the CGRNN for the training and classification. The result demonstrated that even using the short-time hemodynamic fluctuation from a single optical channel, we could achieve a rather high accurate classification with 92.2% accuracy, 85.0% sensitivity, and 99.4% specificity. Receiver operating characteristic curve (ROC) Curves also showed that the performance of CGRNN for the classification between ASD and TD is better than GRU, CNN, and Long Short-Term Memory (LSTM) model, implying that CGRNN is a suitable deep-learning neural network for predicting ASD by using spontaneous hemodynamic fluctuations recorded by fNIRS.

## Materials and Methods

### fNIRS Data Collection

In this study, we used a continuous wave fNIRS system (FOIRE-3000, Shimadzu Corporation, Tokyo, Japan) to record spontaneous hemodynamic fluctuations. fNIRS uses near-infrared light to probe brain activity in terms of HbO_2_ and Hb. As an optical imaging modality, fNIRS is relatively low cost, portability, safety, low noise (compared to fMRI), and easiness to use. Unless EEG and MEG, its data are not much susceptible to electrical noise. At the same time, it can measure the blood flow changes in the local capillary network caused by neuron firings (Naseer and Hong, 2015). FOIRE-3000 has 16 light sources and 16 detectors. Each light source emits three different wavelengths (780, 805, and 830 nm) near-infrared light in an alternating way. The back reflected light which has passed through the cortex is received by neighboring light detectors. Each source-detector (SD) pair forms a detection channel with a fixed SD distance of 3.0 cm. The fNIRS is used to measures the change in light intensity of the three wavelengths, which is converted to the concentration change in hemoglobin (e.g., HbO_2_, Hb, and HbT) by the modified Beer-Lambert law. Neural activity can induce a change in hemoglobin concentration in the local region of the cortex through the neurovascular coupling, which is the basic principle of fNIRS (and fMRI).

Twenty-five children with ASD and twenty-two TD children with an average age of 9.3 (±1.4) and 9.5 (±1.6) respectively were recruited in this study. They were all right-handed. Among them, the ASD group consisted of eighteen boys and seven girls. The TD group included eighteen boys and four girls. Experienced clinicians diagnosed all ASD patients in hospitals. Before fNIRS data collection, each subject was informed about the experimental protocol and written informed consent was obtained from his/her parents. During the data collection, the subject sat in a dark, quiet room with their eyes closed and tried to stay still. The spontaneous (or resting-state) hemodynamic fluctuations were recorded from the bilateral inferior frontal and temporal regions on each subject. The experimental protocol is following the ethical standards of the Academic Ethics Committee of South China Normal University (Zhu et al., 2014). It meets the Helsinki Declaration (Inc, 2009).

**FIGURE 1:**
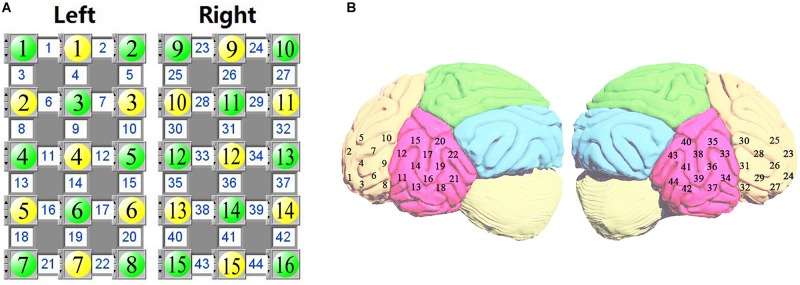
The source-detector configuration where the yellow circles indicate the sources, the green circles indicate the detectors, and the white square between a source and a detector is a channel **(A)**. Location of fNIRS measurement channels over the inferior frontal and temporal cortex **(B)**.

Figure 1A represents the location of fNIRS measurement channels. Yellow circles indicate light sources and green circles represent light detectors. The number (1–44) in the white square is the number for the channel (each channel consists of a pair of a light source and light detector). Figure 1B displays the location of each channel on the brain cortices. The probing area included the bilateral inferior frontal gyrus (1–10 for the left and 23–32 for the right) and bilateral temporal lobe (11–22 for the left and 33–44 for the right). In locating channel positions, the international 10–10 system for EEG was referenced. For each subject approximate 8-minute spontaneous hemodynamic fluctuations were recorded with ∼70 millisecond time resolution, corresponding to a sampling rate of 14.29 Hz.

### Data Analysis

#### Data Processing Flow

**FIGURE 2:**
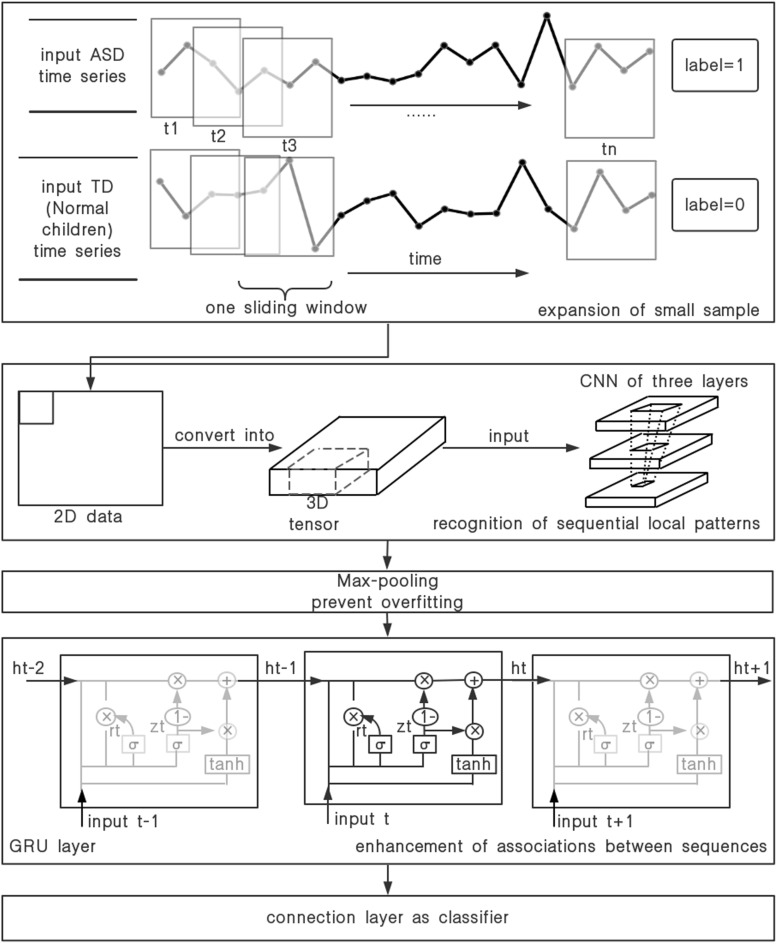
The process of the time-series data.

Figure 2 gives an overview of our data processing flow. The flow divides into two parts. The first part is to increase the sample data for the problem of the small amount of original hemoglobin data. We use the fNIRS time series data as input, and traverse the ASD and TD raw hemoglobin data in the form of sliding window. Each data set can be transformed into a series of continuous and partially overlapping sub-sequence. Each sub-sequence is the data within a labeled sliding window. Hence, the expansion of the small sample data set is performed. The second part, we propose a multilayer neural network, CGRNN model, which combines CNN and GRU, and demonstrate its utility on the accurate classification between ASD and TD with a short-time fNIRS time series. The function in each part of the model is listed below:

(1) The first part of the model uses three-layer CNN to complete the local mode recognition of fNIRS time series. CNN can extract local sub-sequence from the input sequence. Its primary process is to perform the equivalent input transformation on each sub-sequence. So the pattern learned from a specific position of the series can be recognized at any other place later. Thus we can complete the identification of the regional pattern of the sequence and strengthen the generalization ability of feature recognition.
(2) The second part of the model adds the max-pooling layer to prevent over-fitting. Max-pooling layer compresses the data in the form of down-sampling to reduce the parameter information and avoids over-fitting. Moreover, it extracts the maximal value of the feature sequence and further excavates the intrinsic characteristics of data.
(3) The third part of the model utilizes GRU to enhance time series association. We make the feature sequences extract by max-pooling as an input of the GRU. The GRU layer is presented in Figure 2. The reset gate (*R*_t_) and the update gate (*Z*_t_) in the GRU are used to capture short-term and long-term dependencies in the sequence. Therefore, GRU can remember the features in the order of time and infer results from features, which serve to strengthen the correlation of series.
(4) The fourth part is the construction of classifier. The distributed feature learned by the full connection layer maps to the sample tag space.

#### Expansion of Small Sample

**FIGURE 3:**
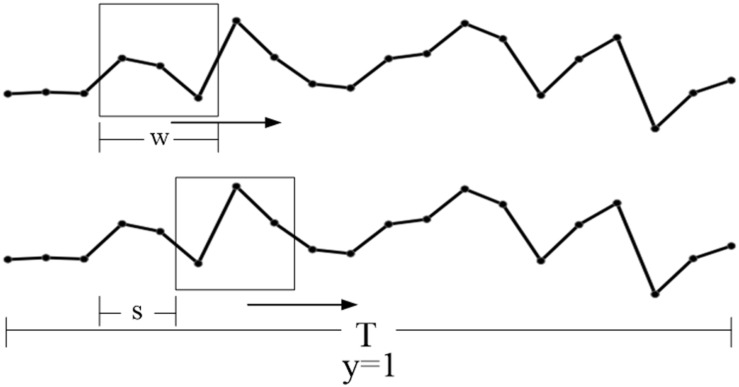
The set of the sliding window (w) and step size (s).

Because of the less number of fNIRS time series, over-fitting is easy to occur in the training of CGRNN model. Thus we expand the data set in the form of setting the sliding window and excavate the distinctive features of the small sample data set further, which maximize the predictive ability of the CGRNN. More specifically, the time-series of one attribute define as “m” in one channel hemoglobin data. One uses a sliding window with step “s” and width “w” (s < < w) to divide the time series, and obtains N = ł(m−w + s)/sł (m > > w) sub-sequences of length w where the symbol “łł” represents the rounding toward minus infinity. Finally, m divides into N overlapping sub-sequences. And the set of sub-sequence is T. There are T = {T_1_, T_2_,.. T_N_}. If the original information is ASD, the sub-sequence collection label is 1. If the original data is TD, the sub-sequence set label is 0. The process of sliding window is shown in Figure 3.

#### CGRNN Model

The CGRNN model is proposed by a combination of CNN and GRU. Among them, CNN is responsible for the identification of the local mode of the original hemoglobin time series, which uses its translation invariant property to extract subtle but distinct features from fNIRS signals, distinguishing the heterogeneity characteristics of ASD from TD in various feature combinations. We use CNN to improve the CGRNN prediction ability. Specifically, first of all, to learn the spatial hierarchies of hemoglobin, the original data is converted into the three-dimensional tensor (samples, time, feature). Then the three-dimensional tensors are put into CNN part for training. CNN uses the convolution kernel to perform the same input transformation for the input data. Local sub-sequences can be extracted from the entire sequence. Patterns learned from one location in the series can be identified at any other location afterward.

Our network uses three convolution layers to introduce the special hierarchical structure of the space filter by making the continuous convolution layer window grow larger. Further, Rectified Linear Unit (RELU) has the function of making some neurons lose activity and reducing the complexity of network structure. Hence, each layer of CNN aggregates a RELU. Finally, the max-pooling layer is utilized to reduce the occurrence of over-fitting further and extract the maximum value of distinctive features by down-sampling. In other word, the data are compressed to reduce the parameter information and excavate the useful information further.

The original time series is prone to gradient disappearance in the course of CNN training, which results in the invalid training. So we use GRU behind the max-pooling layer to solve this problem. GRU network model is a sequence structure that prevents the gradual disappearance of early information by carrying information across multiple time steps. It mainly contains two gate functions (reset gate and update gate). The reset gate and the update gate are utilized to capture the short-term and long-term dependence in the sequence, respectively. GRU remembers the features in the order of time and infers results from features, which serves to strengthen the correlation of series. The specific calculation formula is as follows:

(1) $${\text{Z}}_{t}=\sigma \,\left({\text{W}}_{\text{z}}•\left[h_{t-1},x_{t}\right]\right)$$

(2) $$r_{t}=\sigma \,\left({\text{W}}_{r}•\left[h_{t-1},x_{t}\right]\right)$$

Where *h*_*t–1*_ is the hidden state at time *t*–1, *x*_*t*_is input. W stands for weight. Z_*t*_ represents the update gate, which determines how much previous information is retained. *r*_*t*_ stands for the reset gate. It provides a mechanism to discard past implicit states that have nothing to do with the future, i.e., reset gate determines how much past information has been forgotten. The activation function of both gates is the sigmoid function with a range of {0, 1}.

(3) $${\tilde{h}}_{t}=\tanh\left(W•\left[r_{t}*h_{t-1},x_{t}\right]\right)$$

(4) $$h_{t}=\left(1-z_{t}\right)*h_{t-1}+z_{t}*{\tilde{h}}_{t}$$

${\tilde{h}}_{\text{t}}$ denotes candidate hidden state. It uses the reset gate to control the inflow of the last hidden state that contains the past information. If the reset gate is approximately 0, the previous implicit state will discard.

*h*_t_ is the hidden state at time *t*, which uses update gate *Z*_t_ to update the last hidden state *h*_*t–1*_ and candidate hidden state ${\tilde{h}}_{\text{t}}$. Update gate controls the important degree of the implicit state of the past at the current moment. If the update gate is approximately 1, the former implicit state will be saved and passed to the present moment.

**FIGURE 4:**
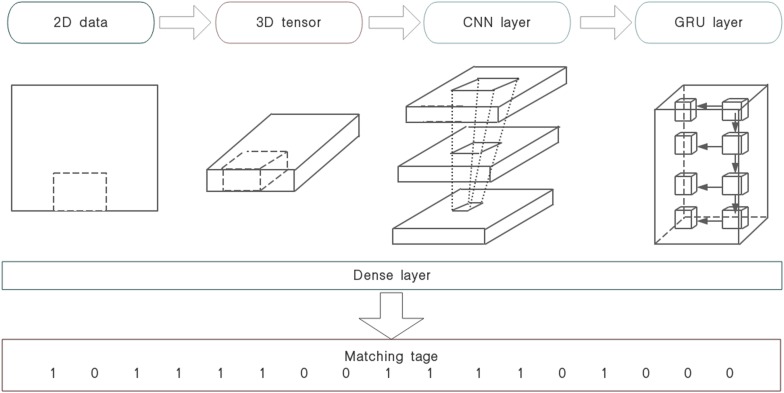
CGRNN flow.

This design can deal with the gradient attenuation of CNN and capture the widely spaced dependencies in time-series better. Finally, CGRNN model builds a classifier in the form of adding a full connection layer on the end of GRU. The distributed feature of learned by the full-connection layer maps to the sample tag space. The CGRNN flow is illustrated in Figure 4.

## Results

### Analysis of Different Channels

To prevent training samples too few to fitting, we adjusted the experimental parameters and divided the data by the sliding window of 100 and step of 50 finally, i.e., considered 7-second data as a sub-sequence. Each sub-sequence corresponded to a specific label. Then we converted the processed data into the three-dimensional tensor (3552, 100, and 1) for the input. We used the data of 28 people to train model. Most of people were divided into 136 sub-sequences. But for a few people the number of sub-sequences was less than 136 because of the recording time was shorter than 8 min (e.g., some children could not tolerate 8-minute measurement, so we had to stop fNIRS recording early). So the final sample size was 3552. Our CGRNN network used three convolution layers. The three convolution layers, respectively had 32, 64, and 128 filters, their kernel_sizes were all five. In each convolution layer, the filter transforms the matrix of a child node in the current layer into the matrix of a unit node in the next layer. The node matrix processed by the filter is determined by the filter size, namely kernel_size. Among of them, the filter slides at regular intervals on the neural network matrix of the current layer and does dot product. In other words, the element of the filter at each position are multiplied by the corresponding element of the input sample and we add up the overall result. We assume that the input sample is [a, b, c, d], and one of the filters is [2, 3], the interval is 1. Then we can get [a × 2 + b × 3, b × 2 + c × 3, c × 2 + d × 3] through the calculation of dot product. The result is called feature map, and the number of feature map is the same as the number of filters. For example, the first convolution layer uses 32 filters, 32 filter maps are obtained after convolution calculation and serve as the input of the second convolution layer. The input of GRU includes the input sample at current time and the hidden state of the previous sample. The hidden state of the previous time and the current time is multiplied by the weight matrix. Then the added data are sent to the update gate, that is, multiply by the sigmoid function. Therefore, the update gate determines how much previous information and current information is retained. The operation of reset gate is similar to update gate. However, the weight matrix of the reset gate is different from that of the update date because the reset gate determines how much past information has been forgotten it. So GRU not only capture the short-term dependence, but also capture the long-term dependence in the sequence. Furthermore, the CGRNN model were trained using the RELU active function, the binary_cross-entropy loss function, and the Adam optimizer with the default parameter values. The dropout rate during training was fixed to 0.5. Learning rate was 0.01. Models were initialized using default settings. The output were 128 filters, each filter was an 88 dimensional vector. Then, the hemoglobin data were divided into three parts: training set, validation set, and test set. Their proportion is 3:1:1. We trained the CGRNN in the training set, evaluated the generalization ability of the model in the verification set, and saved the optimal model with the smallest loss function. Finally, the model was tested on the test set.

**FIGURE 5:**
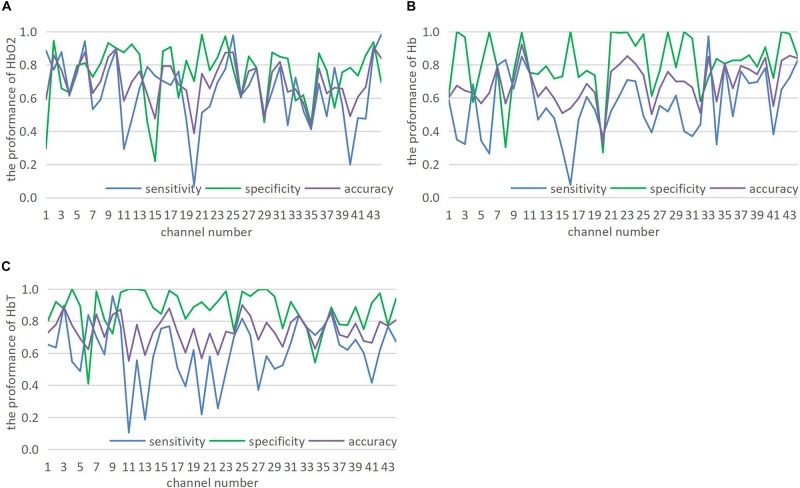
**(A–C)** Show the prediction of 44 channels in HbO_2_, Hb, and HbT three attributes.

**FIGURE 6:**
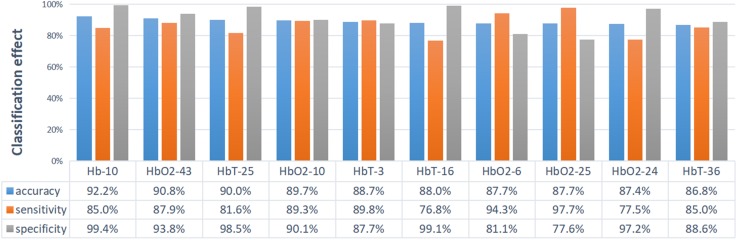
The display diagram of the top 10 channels with corresponding properties that have the best classification effect. Ten sets of data are sorted from large to small by accuracy.

To evaluate the performance of CGRNN classifier, this study applies sensitivity, specificity, and accuracy to the test results of CGRNN classifier (Cheng et al., 2019). Among them, ASD calls positive class, TD calls negative class. Sensitivity means the proportion of actual positives that are correctly classified in all positives. Specificity is the proportion of actual negatives that are correctly identified in all negatives. Accuracy defines the percentage of correct diagnoses among all diagnoses. More specifically, we consider testing every 7-second data of one attribute with a single-channel. There are 44 channels for each person’s data in the test set. Each channel consists of three attributes (HbO_2_, Hb, and HbT). Each attribute divides into sub-sequences in the form of the sliding window. Every sub-sequence of 7 s tests its sensitivity, specificity, and accuracy. Finally, the average of sub-sequences denotes the final sensitivity, specificity, and accuracy. The test results are presented in Figures 5, 6.

The hemodynamic activity recorded from the bilateral inferior frontal and temporal lobes vary significantly, which may imply that not every location (or channel) is closely associated with ASD, or sensitive enough for the discrimination, so it is not necessary to achieve good accuracy for the classification in every channel. If the characteristics of ASD can extract from brain images collected in few channels (which might be closely associated with ASD), it is of great practical significance for brain imaging study in ASD.

Figure 5 shows that CGRNN classifier performs well on HbO_2_, Hb, and HbT. Although the classification results of different channels under the same attribute are quite different, some channel classification effects are significant. Therefore, CGRNN classifier perform an accurate distinction between ASD and TD children. Moreover, though HbT is the sum of HbO_2_ and Hb, it may provide richer discriminative information than HbO_2_ and Hb. For instance, the accuracy of HbO_2_, Hb, HbT in channel three are 76.8, 64.4, and 88.7%, respectively.

Accuracy represents the overall diagnostic accuracy of ASD and TD. Hence, Figure 6 sorts the accuracy from large to small and shows the top ten channels with their corresponding attributes. The classification effect is evaluated by accuracy, as shown in Figure 6. The first is the Hb of channel 10: 92.2% accuracy, 85.0% sensitivity, and 99.4% specificity. The second is the HbO_2_ of channel 43: 90.8% accuracy, 87.9% sensitivity, and 93.8% specificity. The third is the HbT of channel 25: 90.0% accuracy, 81.6% sensitivity, and 98.5% specificity. Most of the functional imaging modalities such as fMRI, MEG, and SPECT, et al., is that the imaging data usually requires recording and analyzing for several minutes. However, the CGRNN model proposed in this paper performs better classification effect by using only 7-second data and has practical application value.

### Classification Effect of CGRNN

**FIGURE 7:**
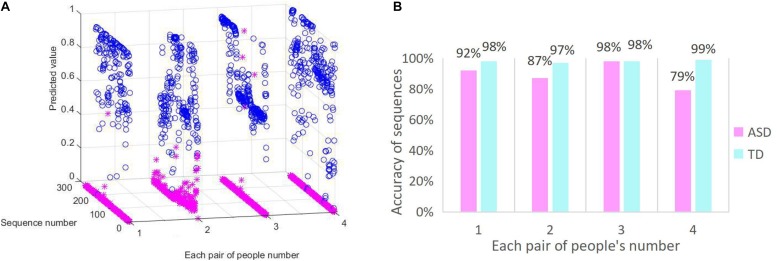
CGRNN classification effect. **(A)** The predictive distribution of test data. **(B)** The accuracy of sequences diagnosis.

Moreover, to visualize the classification performance of the CGRNN model further, we randomly select four pairs of ASD and TD children to display the results of the test data. To expand the number of test samples and make the predictive result more accurate, we splice together the data from the same column. Specifically, for each people, we splice 6,859 rows of one column data into 13,718 rows of one column data. Then the time series for most of people is divided into 273 sub-sequences by using a sliding window with a width of 100, and a step of 50. The predictive result for each sub-sequence is displayed in Figure 7A. The average of the accuracy is shown in Figure 7B.

The classification effect of ASD and TD children is illustrated in Figures 7A,B. Figure 7A is the real distribution of test data. The *X*-axis represents the randomly selected four pairs of people in the test set. Each pair consists of an ASD child and a TD child. ASD children express by blue, and TD children express by pink. *Y*-axis represents a collection of sub-sequences. *Z*-axis represents the predicted value of CGRNN model. Predictive values range from 0 to 1. The predictive value of TD children is less than 0.5 for accurate forecasting, and the predicted value of ASD is more than 0.5 for precise prediction. Predicted values of ASD data are represented by “o.” The Others are represented by “^∗^.” Figure 7B shows the diagnostic accuracy of sub-sequences. The *X*-axis represents the four pairs of people randomly selected in the test set. The *Y*-axis is the percentage of correctly diagnosed sub-sequences in the whole sequences.

We can see that the predictive values of ASD are basically between 0.5 and 1.0 from Figure 7A. Most of the predicted values of TD concentrate in the vicinity of zero. So there are four clear lines on the *Y*-axis, and these are stacked by “^∗^.” In other words, most of the TD test results are correct. As shown in Figure 7B, the average predictive value of ASD children greater than 0.5 is 89% and the average predictive value of TD children less than 0.5 is 98%. Because each sub-sequence is a 7-second fNIRS time series, we can assume that the recognition accuracy of 7 s of ASD data is 89%, and the recognition accuracy of 7 s of TD data is 98%. Therefore, the CGRNN model can effectively distinguish between ASD and TD children in a short time (7 s).

Since our model is the first ASD classification using a single channel of fNIRS time series data that employs neural network model, there are no canonical comparison partners. We thus compared our model with some widely used traditional classification algorithms such as Logistic Regression (LR), k-Nearest Neighbor (KNN), Random Forest (RF), and Support Vector Machine (SVM) classification methods. LR is a generalized linear regression analysis model, it is often used in the binary classification of disease diagnosis. KNN is a well-known machine learning classification algorithm, it determines the category of the sample to be divided according to the category of the nearest sample or samples. RF is a classifier that uses multiple trees to train and predict samples, its classification performance is much better than LR and KNN algorithm. With limited sample size, SVM has stronger ability of generalization in comparison with other existing machine learning algorithms. Firstly, we divided data set in the form of the sliding window (*w* = 100, *s* = 50), the input to these models were two-dimension data (the number of samples, window_size). Then we used GridSearchCV to tune hyper parameter and made feature engineering. By contrast, the CGRNN model outperformed the other four traditional models as shown in Table 1.

**TABLE 1:** Accuracy of different classification models.

**Model**	**LR**	**KNN**	**RF**	**SVM**	**CGRNN**
Accuracy	61.5%	65.0%	80.2%	81.2%	92.2%

## Discussion

### ROC Comparison Under Different Neural Network Models

**FIGURE 8:**
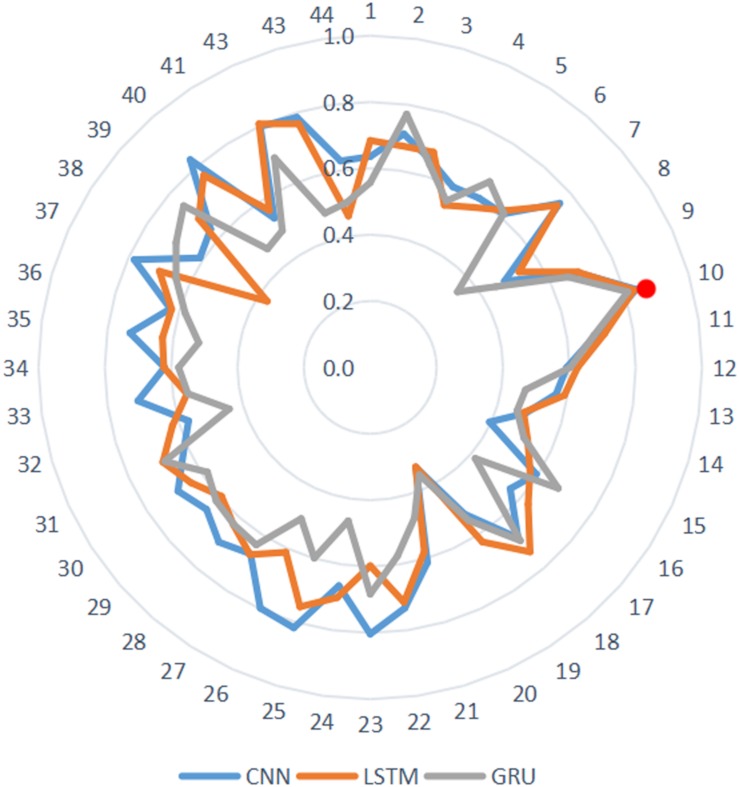
The performance of 44 channels.

For further determining the efficiency of our model, we respectively, applied CNN, LSTM, and GRU to the classification between the children with ASD and TD children based on short-time spontaneous hemodynamic fluctuations. CNN is a feedforward neural network, it not only plays an important role in computer vision tasks, but also has an impact on the time-series analysis. CNN can extract features from local time-series data by using convolution, modularize represented information, utilize data more efficiently. LSTM can overcome the limitation of vanishing gradient in time-series analysis of RNN, thus can capture long-term dependence in time sequential learning. Compared with LSTM, GRU has one less gating unit, which leads to fewer parameters and easy convergence, its computation cost is lower. In these neural network models, the same input data sets as in the CGRNN model were used. The best parameters for each model was selected by validation. We tested the accuracy of each model in 44 channels, and displayed the results in Figure 8. The red dot shown the four neural network models (CNN, LSTM, and GRU) also have good performance in the classification for channel 10.

Then we further used ROC to aggregate characteristics of “True Positive Rate” (TPR) and “False Positive Rate” (FPR) and evaluate CGRNN model classification by comparing with ROC curves of different models. TPR represents the proportion of true positives that are correctly classified in all true positives. FPR denotes the percentage of false positives that are correctly classified in all false positives. Empirical ROC curve takes TPR and FPR as ordinate and abscissa, respectively. The TPR and FPR points show different diagnostic locations. These are connected to compose ROC curves. Without considering the effect of misdiagnosis and missed diagnosis, we make the diagnosis point closest to the top left corner (0, 1) as the cut-off point (Fawcett, 2005).

**FIGURE 9:**
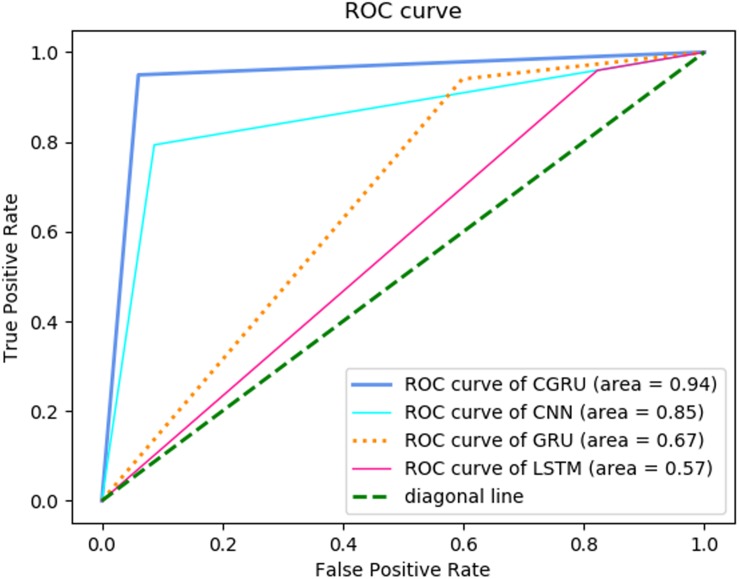
ROC comparison for different models.

Figure 9 showed the comparison of ROC curves of different models. Since hemoglobin data of channel 10 had the best-classified effect, we selected channel 10 to respectively, verify the ASD classified effect of CGRNN, GRU, CNN, LSTM four different neural network models. Each test selected three thresholds, i.e., max value, min value, and mid-value. Each threshold corresponded to a point (FPR, TPR). All coordinate points were connected to draw the ROC curve these points were used to identify different algorithm performance visually.

CGRNN model was closest to the upper left corner in the ROC curve, so it had the best classification effect. The second was the CNN model, which was helpful to extract hemoglobin features. The third was the GRU model, which cannot play a useful role in classification. Finally, the LSTM model had the worst classification effect. Besides, we compared the area (AUC) under the ROC curve of each model. AUC is a comprehensive measure of all possible classification effect. It regards as the probability that the model randomly arranges the positive sample above negative sample. Generally speaking, the larger the AUC value is, the better the classification effect is (Fawcett, 2005). The comparison shown that the AUC of GRU algorithm was the largest. Therefore, the diagnosis of CGRNN algorithm is the most valuable.

Furthermore, most of the previous studies utilized multiple feature variables to perform effective differentiation between ASD and TD. No one applied seven seconds’ data of single-channel to achieve a better classification effect. The CGRNN uses the 7-second test data of the Hb attribute of channel 10 to have 92.2% accuracy, 85.0% sensitivity, and 99.4% specificity. Therefore, the CGRNN model cannot only diagnose patients with autism efficiently and accurately but also avoid the misdiagnosis of healthy people.

### Comparison of the Classification Ability of Brain Regions

**FIGURE 10:**
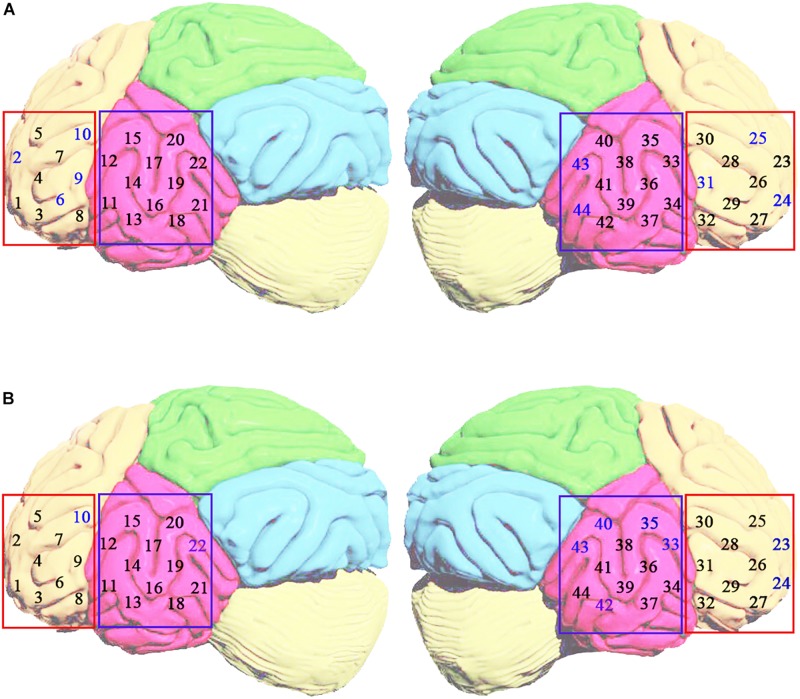
The distribution of best classification performance (i.e., accuracy >80.0%) channels (blue number) based on HbO_2_ **(A)** and Hb **(B)** attributes in the left and right brain regions. The yellow area indicates the frontal lobe. The rose-red area represents the temporal lobe.

Figure 10 displays the location of selected channels with good differentiating ability (accuracy >80.0%) in the HbO_2_ and Hb attribute. For HbO_2_ (Figure 10A), there are seven channels locating in the frontal lobe (4 on the left, 3 on the right), and two channels situated in the temporal lobe (both on the right), indicating that the HbO_2_ data of the frontal is more discriminative than the temporal area. Among them, the most discriminative channel 10 locates in the left frontal region. Overall, on the HbO_2_, the data from the left and right hemisphere has little difference in classification ability (four channels on the left and five channels on the right). For the Hb data (Figure 10B), there are seven channels on the right and two channels on the left, indicating that the Hb data from the right brain is more separable.

## Conclusion

Our study aims to explore the feasibility of using a multilayer artificial neural network for the classification between children with ASD and TD children based on short-time spontaneous hemodynamic fluctuations.

The contribution of this study has three aspects. First of all, a multilayer neural network called CGRNN was used, which combined three-layered CNN and one-layered GRU. Since CGRNN has a strong ability in finding characteristics associated with ASD and acquiring intrinsic relationship in fNIRS time-series, it can accurately predict ASD by using a short fNIRS time series, which is of great significance for brain imaging research on ASD.

Secondly, different from using small sample data of fNIRS, we expanded the data in the form of the sliding window and combined the CGRNN model to excavate the intrinsic characteristics of the data and improved its predictive ability. The result showed our model performed better than the other four traditional algorithms such as LR, KNN, RF, and SVM. Furthermore, we used ROC curve to compare our model with CNN, LSTM and GRU neural network model to demonstrating the reliability of our model.

Finally, we demonstrated that though HbT is the sum of HbO_2_ and Hb, it may provide richer discriminative information than HbO_2_ and Hb. On HbO_2_ attribute, the hemodynamic signal from the frontal lobe rather than the temporal lobe leads to a better classification. On Hb attribute, hemodynamic signal from the right hemisphere contains more discriminative information between ASD and TD than the left hemisphere.

## Data Availability Statement

The raw data supporting the conclusions of this manuscript will be made available by the authors, without undue reservation, to any qualified researcher.

## Author Contributions

LX designed the outline of this study and analyzed the data. XG and XH contributed to writing and modifying manuscript. JY participated in the logical construction and design of study and supervised the whole process. JL analyzed the feasibility of the article from a perspective of fNIRS brain imaging and revisited the manuscript critically. All authors contributed to manuscript revision, read and approved the submitted version.

## Conflict of Interest

The authors declare that the research was conducted in the absence of any commercial or financial relationships that could be construed as a potential conflict of interest.
